# Co-creation and engagement in a DNA integrity cohort study

**DOI:** 10.1017/cts.2023.556

**Published:** 2023-05-22

**Authors:** L. Lynette Parker, Chantel M. Bonner, Robert W. Sobol, Martha I. Arrieta

**Affiliations:** 1 Center for Healthy Communities, Frederick P. Whiddon College of Medicine, University of South Alabama, Mobile, AL, USA; 2 Mitchell Cancer Institute, University of South Alabama, Mobile, AL, USA; 3 Department of Pharmacology, College of Medicine, University of South Alabama, Mobile, AL, USA; 4 Department of Pathology and Laboratory Medicine, Warren Alpert Medical School & Legorreta Cancer Center, Brown University, Providence, RI, USA

**Keywords:** Community Advisory Board, study recruitment, DNA integrity, underrepresented populations, community-engaged research

## Abstract

**Introduction:**

The partnership between a research community engagement team (CE Team) and a community advisory board (CAB) formed the basis for bidirectional communication in developing resources for participant recruitment in a DNA integrity study. Engaging with a minoritized community, this partnership focused on respect, accessibility, and expanded engagement.

**Methods:**

A ten-member CAB, working in two groups defined by meeting time convenience, provided insight and feedback to the CE Team in the creation of recruitment and consent materials, via an iterative design process in which one CAB group reviewed and enhanced materials, and the second group tested and refined them further. The continuous analysis of CE Team notes from CAB meetings captured information needed both for materials refinement and implementation of CAB-suggested activities.

**Results:**

The partnership resulted in the co-creation of recruitment and consent materials that facilitated the enrollment of 191 individuals into the study. The CAB encouraged and assisted in expanded engagement inclusive of community leaders. This broader engagement provided information about the DNA integrity study to community decision-makers as well as responded to questions and concerns about the research. The bidirectional communication between the CAB and the CE Team encouraged the researchers to consider topics and research interests related to the current study but also responsive to community concerns.

**Conclusions:**

The CAB helped the CE Team develop a better understanding of the language of partnership and respect. In this way, the partnership opened doors for expanded community engagement and effective communication with potential study participants.

## Introduction

In 2015, the National Institute of Environmental Health Sciences hosted the meeting “Workshop on New Approaches for Detecting DNA Damage and Mutation in Population Studies.” Workshop participants agreed that the field of DNA damage, repair, and mutagenesis had the potential to contribute to the development of strategies for personalized disease prevention. They stressed that population-based studies, including members of diverse communities, were needed to develop the understanding and technologies to reach this level [1]. However, minoritized communities experience many barriers to participating in medical research, which are amplified by aspects of working with genomic data [2,3].

Mistrust, based on historical memories of abuse and marginalization, creates skepticism about the purpose of research studies as well as a fear that scientists will ignore the individual rights and needs of research participants in their pursuit to further the goals of science [4–6]. The distrust of both research and the medical community opens concerns of mistreatment in terms of research benefitting one race over another with people of color being left out [7–9] as well as concerns that protections for confidentiality and the privacy of medical information are not respected [3,5,10].

Fear of the misuse of genetic information adds another set of barriers to research participation from historically marginalized groups. For some community members, DNA-related studies open up the participant and their relatives to investigation if law enforcement agencies gain access to the research samples [3,7]. A related concern is the possibility of genetic discrimination by employers and insurance companies if privacy and confidentiality are not maintained [3,5,7]. Many community members believe that signing an informed consent form causes a loss of individual rights [7]. A lack of a common language to bridge the gap between research teams and community members contributes to the fear and misconceptions about genetic research [3,11].

Community engagement provides principles and values for addressing the barriers to minority participation in genomics research. It focuses on building relationships between academic investigators and community members to develop bidirectional communication yielding increased investigator understanding of the community context, experiences, and concerns, while also expanding community understanding of research processes, terminology, and protections to promote more trust and confidence in medical research [4,12–14]. It creates a pathway to improve research studies through the sharing of research procedures and materials, exploring how those may cause confusion, and working with community partners to edit materials and processes to improve communication with possible research participants [15]. Likewise, the informed consent process can be strengthened in ways that build knowledge about genomics research and equip community members to make more confident decisions about study participation [11,14]. By improving study design and tools, community engagement offers opportunities for increasing diverse participation in genome-related research, while also improving dissemination and translation of findings into impactful interventions [4,13].

Community Advisory Boards (CABs) represent one mechanism for establishing the desired community-investigator dialog. CABs consist of individuals representing communities that meet regularly with research teams to discuss study protocols and activities [13]. They bring the community voice to research ventures by providing insights into community culture and interests, advocacy for the rights and needs of historically marginalized communities, and local expertise on the development of more accessible study materials [8,12,15]. Through their partnership, CAB members and investigators can develop a deep respect for each other, an understanding of the needs of each, and a focus on ensuring that research will benefit those from groups experiencing disadvantages and not just academic goals [7,16].

In this article, we describe the experience and outcomes of a community engagement team (CE Team) and CAB partnering to develop recruitment processes for a DNA integrity study implemented in a primarily African American community. We share lessons and practices that helped operationalize recruitment and enrollment for the study. The recruitment and implementation of the CAB as well as all procedures for participant recruitment and data collection for the DNA integrity study were reviewed and approved by the Institutional Review Board of the University of South Alabama (USA).

## Materials and Methods

### CE Team

The DNA integrity study involved a partnership between a genomic science lab and a community-engaged health equity research center operating within USA’s Health System and College of Medicine, respectively. The three-member CE Team was responsible for implementing community informed recruitment and retention of study participants, as well as data collection. The Lab Team performed all DNA integrity measures on lymphocytes isolated from blood samples provided by the participants [17]. The CE Team has a long history of community-engaged work, comprising research, health advocacy, and promotion of diversity within the health sciences [18–23].

### Study Sample

The source population (*n* = 115,633) were residents of 11 zip codes comprising communities whose members are underrepresented in biomedical research (UBR) [24]. Following a snowball sampling design [25,26], the CE Team initially invited members of an UBR cohort who had previously participated in a four-year project to document progression of health equity in the study area. We were able to contact 51 (44.7 %) former cohort participants. Those who decided to participate in the DNA Integrity study (*n* = 19) were asked to refer relatives or friends the CE Team could reach to provide information about the study and invite for participation. Many of the persons referred were residents in areas beyond the initial zip codes. We eventually expanded the residence inclusion criteria to encompass 20 zip codes. Based on lab processes considerations, the sample size for the study was set at 240 participants.

### CAB Description

The CAB consisted of eight women and two men of African American descent recruited from various groups working with the CE Team, all of them stakeholders within the communities comprising the initial source population: five CAB members are Community Health Advocates [21,22] (two retired nurses, a community activist, and a social worker), all of whom have organized community health events for several years. The remaining five members comprised the CEO of a community-based organization focused on community health and development, two community members, a health care provider overseeing a low-cost clinic in one of the disinvested communities covered by this project, and the pastor of a church located in the same area.

In terms of CAB member research knowledge and training, the two community members previously worked as Research Apprentices [20] for the CE team and had been introduced to the basics of research participant protections, survey methodologies, data management, and analysis. The pastor graduated from the Community Research Fellows Program [27] offered by the Gulf States Health Policy Center in which he was exposed to research ethics and methodologies. The CEO is a long-standing partner of the CE team and has a fairly extensive knowledge of community-engaged research as well as of the rights of persons participating in research. CAB members received a $100 monetary incentive to participation in each meeting. They are acknowledged in all publications and activities related to the study.

### Iterative Design

The CAB met 13 times between March and August of 2019 to design recruitment and informed consent materials. Each meeting lasted two and one-half hours. In response to CAB member realities, meetings were scheduled for both Wednesday and Saturday mornings with members able to choose which best fit their needs. In practice, CAB members tended to be consistent in the days chosen resulting in two established groups that built a comfortable atmosphere for engagement and input, with subsequent Wed and Sat meetings occurring approximately once per month. This consistency in groupings facilitated a natural environment for the iterative process of discussion, creation, testing, revising, and finalizing procedures as well as study materials [28–30].

The CE team prepared an agenda and objectives for each meeting building on previous discussions while introducing new or related topics for further examination. The agendas varied between the two groups with lessons from the Wednesday CAB meeting incorporated into the agenda for the next meeting of the Saturday CAB group. Throughout the meetings, the discussions centered around five guiding concerns: building understanding, communicating in plain language, procedures to interact with possible participants, community notification, and future orientation building a foundation for developing broader research understanding in the community.

During CAB meetings, a member of the CE team took official notes in an electronic format. Note keeping focused on capturing as much of the conversation as possible, especially CAB opinions, ideas, and stories related to the recruitment materials and the study in general. After each meeting, these notes were finalized in a progressive format, where each participating CE team member, in turn, read the official notes and added data, notes, and commentary to the official record. The CE team then held debriefing sessions consulting the record to garner CAB meeting takeaways, how these should be implemented, and steps for moving forward. This process engendered a rich understanding of what took place in each meeting, enabling the team to quickly implement suggestions, learn from the CAB members’ experiences, and prepare objectives and agenda for the subsequent CAB gathering. In including verbatim quotes within the manuscript, they are identified using the study year, the meeting number, and the first letter of the day in which the meeting took place.

## Results

From the beginning, the CAB pushed the CE Team to think in terms of the neglect and abuses experienced by African American communities in research studies, with specific references to Henrietta Lacks and other examples of maltreatments, warning of both the possibility of repeating past abuses and of causing distress in the community. The CAB put forward questions and concerns about the study before contributing to practical considerations and actions, as seen in the following quote from a female CAB member in her 60s *“We have to overcome the past histories of research impact on African Americans. That’s why you need to present what you are doing and how you are going to handle this. Acknowledge you know this history.”* ^(Y1M4S)^ Further, throughout the process, the CAB stressed the importance of communicating in a respectful manner, explaining that *“People feel that they are not respected in research, so we need to change it.* [Language in retention script] *sounds more respectful.”* ^(Y2M5W)^

CAB interactions offered valuable insights and concrete action steps to move the DNA integrity study forward, leading to the recruitment of 191 study participants over a three-year period inclusive of a seven-month pause due to COVID lockdowns. Recruitment was capped at 191 when the Lab Team realized they had enough samples to complete the DNA integrity analysis. Ninety-six percent of those recruited were of African American descent, two-thirds identified as female, and one-third as male. Close to half (47.5 %) of the participants were 45–64 years of age, a third were 19–34 years old and 19.4% were 65 years or older.

**Figure 1.**
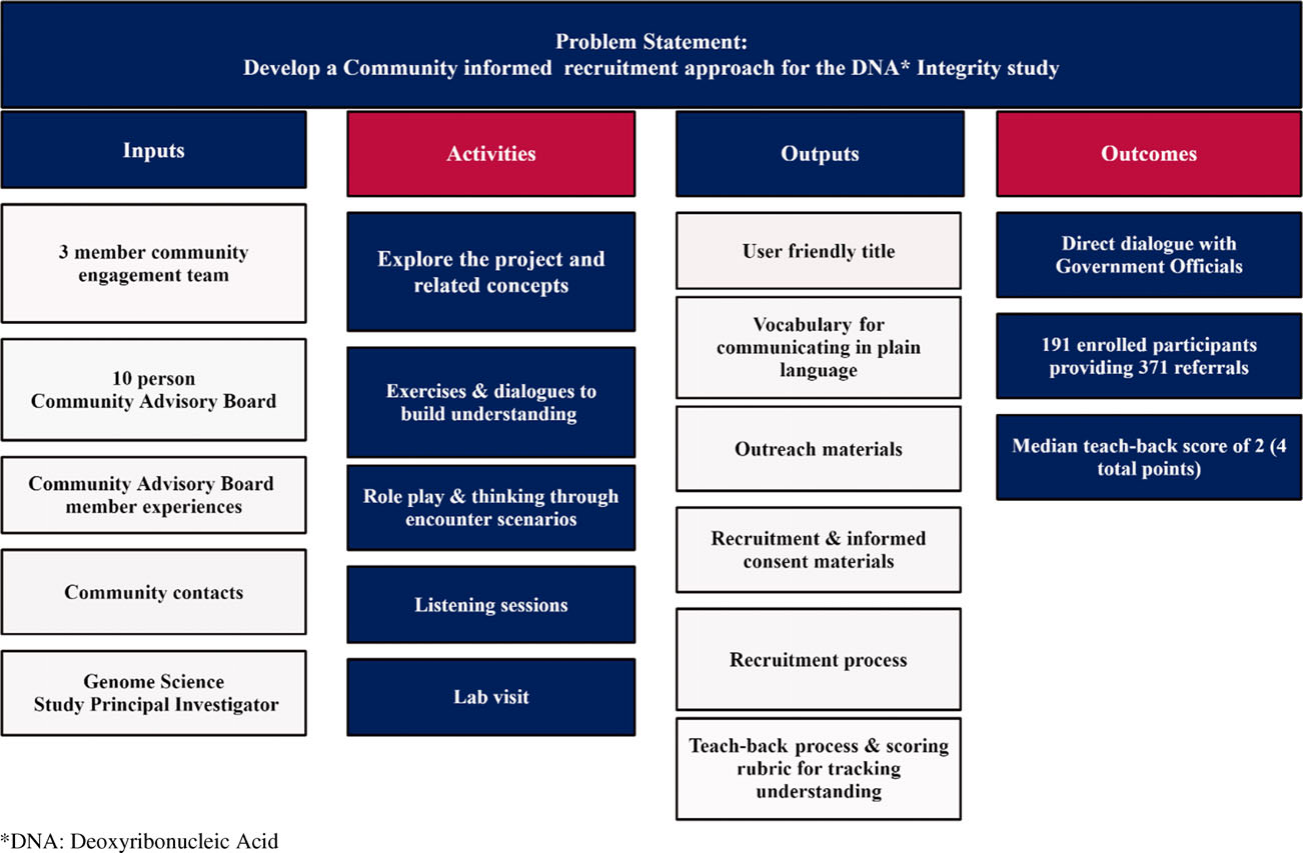
Retrospective logic model of results.

The CAB–CE team partnership results fell into three broad categories: co-creation of materials and processes, expanded community engagement, and bidirectional communication. Fig. 1 is a retrospective logic model of the CAB process.

### Co-creation

CAB–CE Team dialogue resulted in the joint development of study processes and materials grounded on an attitude of respect and humility and the use of vocabulary that prioritized plain language. The vocabulary discussion started with the development of a “user-friendly” title by deconstructing phrasing in the official project title (“Measuring genomic DNA damage and DNA repair capacity in longitudinal population samples – a step towards precision prevention”), defining terms, and developing better terms (see Table 1).

**Table 1.** Thinking through understandable language

Original Phrase	Community Advisory Board Consensus	Language to Use
Genomic DNA* Damage	“DNA is known in the community, but the word “genomic” is confusing.	Use DNA only and discuss its function in the body.
DNA Repair Capacity	Ability to heal is easier to understand	DNA healing
Longitudinal Population Sample	Overtime in the same group of people; drop from the title.	Two-year return for a second blood sample
Precision Prevention	Prevention is understandable. Precision does not add to the understanding.	Disease prevention

* DNA: deoxyribonucleic acid.

The analysis and associated consensus-building activities (e.g., successive rounds of voting on suggested titles) resulted in the user-friendly title “DNA Healing and Disease Prevention.” The deep discussion about language and meaning fed into the development of other resources and processes.

### Outreach Process and Materials

Focusing on expediency to identify and recruit former cohort members, the CE Team suggested a three-step process: (1) a visit to the homes of previous participants with a study flyer, (2) for those not contacted at the home visit, attempted contact via phone calls, and (3) for those not reached by phone, an attempt to reach through regular mail via a postcard.

In leading the decision-making, the CAB redesigned the outreach process to effectively shift the control to the potential participant’s side. They recommended to start by providing relevant information to allow the possible participant to make decisions about interacting or not with the research team, via an invitation postcard including the name of the study, a mention of the monetary incentive – but not the amount – and contact information to learn more. This was followed by three contact attempts by telephone (using whitepages.com to find more up-to-date contact information) and a final letter.

The CAB diligently reviewed and edited the printed outreach materials, formulating an attractive design for the invitation postcard, courteous and non-judgmental wording for the final letter, and redesigning a factsheet to be mailed with the final letter. In the latter case, the CE Team produced a draft that included five questions: (1) What is the study about; (2) Why am I invited; (3) What will be asked of me; (4) Why should I participate; and (5) How do I learn more. The CAB took exception to the question of “why am I invited?” which focused on the inclusion criteria for the project. They raised concerns such as suspicion of “targeting” in the Africa American community and communicating a utilitarian outlook toward possible participants. The CAB rewrote the fact sheet to collapse questions 2 and 4 into the question “Why am I important to this research?” which comprised the inclusion criteria, the reason the specific individual was being contacted, and how participation would contribute to the goal of the study.

The CAB also provided valuable insight into the design and content of telephone scripts, outlining key points to include in the initial conversation with possible participants: (1) mention the invitation postcard; (2) summarize the study; (3) discuss eligibility criteria; (4) confirm their ability to travel to data collection site; and (5) schedule a face-to-face informational meeting. The CAB also edited a voicemail script created by the CE team.

In each case, the CAB stressed respectful, easily understood language. For example, in creating materials the CE team tended to alternate between the words “project” and “study.” The CAB repeatedly advised the team to only use the word “study” for two reasons: (1) the consistent language would help possible participants more easily connect with the message being communicated, and (2) the word “project” came across as focusing on the participant as a “project” instead of inviting them to be a part of the “study.”

### Recruitment Process and Materials

Recruitment occurred in-person. Members of the CE team met with possible participants who indicated that they wanted to learn more about the research and further consider participation. To develop the joint vocabulary for explaining the study, the CAB brainstormed scenarios and questions that the CE team could encounter. They also coached the CE team on how to approach the interactions. This included full role plays: from initial telephone calls to a mock in-home informational meeting where the invitation to participate would be formulated and the process of signing the informed consent document might be completed. Through these coaching sessions, the CAB worked with the team to simplify language use and reiterated core values of respect, transparency, and authenticity.

Along the lines of simplified communication, the CAB worked with the CE team to write a more easily readable informed consent document. However, the document continued to be nine pages long and presented a complex tiered consent process which included, beyond consent to the study procedures, additional consents to allow the lab to store any blood sample remaining after the DNA integrity measurements were taken to be used in other research. Therefore, visual aids, in the form of two booklets were created to facilitate the informational meeting and informed consent process. The booklets were organized with the same section headings as the informed consent document, using simple language and images to communicate the key concepts presented in explaining the study purpose and procedures.

The first booklet, internally called the project booklet, covers DNA’s function in the body, some ways in which DNA becomes damaged, the mechanism of DNA healing, and the consequences when DNA is not repaired. In terms of the research study, the booklet covers the purpose, procedures, and inclusion criteria. A CAB member highlighted the characteristics and value of the booklet as follows: *“It narrows down the specifics pulling them in; get their attention; not fooling them; giving them what they need to know; feel like owners of it; and it’s accountable to the community.”* ^(Y1M3W)^

The second booklet, used if someone expressed a desire to participate in the study, provided information adapted from the “Research Subject’s Bill of Rights,” the risks and benefits of participating, and contact information for the principal investigators and the university’s IRB. The booklets – edited and finalized through the CAB dialogue – served as an important tool for guiding the informational and consent conversations while providing a reference for participants in asking questions and understanding the concepts in the informed consent document.

The review and discussion of the second booklet opened a conversation about the importance of participant’s rights and the need to recognize them, especially confidentiality. CAB members raised questions of whether or not HIPPA regulations would apply to the data that were collected. They stressed the importance of explaining privacy protections to possible participants. Concerns about confidentiality ranged from *“Make sure the postcard doesn't specify that this person was a previous participant.”* ^(Y1M1S)^ to the serious issues of handling genetic materials, such as not sending DNA data to law enforcement agencies and not allowing the samples to be used in paternity testing. Related quotes are presented in Table 2.

**Table 2.** Excerpts from Community Advisory Board meeting notes regarding rights and confidentiality

Rights and Confidentiality	Quote Identifier
Make sure to emphasize that it [DNA** information] isn't going anywhere else	(Y1M1S)*
Assure them you are not selling it to the FBI*** databases	(Y1M1S)
Not trying to find out if you’re the baby daddy [making this clear to participants is important given what they hear about DNA on Television]	(Y1M3W)
[Rights booklet] One thing people are going to be concerned about. Who are you going to share it with? You say you aren't going to share it. But are they [going to believe it?]…	(Y1M4S)
Are people required to come in every two years? What is the risk to the research if they only come in year one? We need to make it clear that they understand this and that they can stop participation at any time.	(Y1M2S)
Things are different for each person. Telling people they have a choice lets them think they still have control. Make sure they have their own copy of the informed consent document.	(Y1M5W)
I think this booklet addresses their rights upfront so that they can make an informed decision. I like that. It is very clear to me the risks and benefits. I think that is the most appropriate approach. What people don't realize is that you’re still building trust.	(Y1M3SD)

* Quote Identifier consists of the study year, meeting number, and the first letter of the day on which the meeting took place.

** DNA, deoxyribonucleic acid

*** FBI, federal bureau of investigation

The significance of providing participants information about their rights played a key role in the deliberations. The CAB emphasized the need to directly present and address rights in an understandable way, demonstrated in the discussion of how to present information from the Genetic Information Nondiscrimination Act of 2008. The university IRB required a summary handout created by their office be given with each informed consent document. In noting the confusion that the legalistic language used could cause, the CAB advised the CE Team to stress the existence of the act and be prepared to explain it in simple terms.

The recruitment process ended with a set of teach-back questions used in the informed consent process. These questions provided a tool for dialogue between the CE team and potential participants, regarding the study and the rights of persons participating in research. The CE team identified an initial set of questions suggested by the University’s IRB. Upon reviewing them, the CAB pointed out various problems with the language and rewrote the questions to resonate with community members. The CAB also aided in the creation of a scoring rubric to help the team gauge the success of the informed consent conversations. In describing the teach-back purpose, a CAB member who is a community advocate explained the role of the teach-back tool well: *“For me, the goal should be to explain it [the DNA integrity study] enough so they are comfortable with the concept when we finish. If they’re only saying you’re going to pay me for my blood, then we didn't do a good enough job.”* ^(Y1M7S)^

### Engagement

During the co-creation process, the study co-principal investigator responsible for community engagement asked the CAB if persons other than participants should be informed about the study. CAB members were quite vocal about the need to inform community leaders and institutions such as churches.

When asked to prioritize, the CAB focused on elected officials representing the zip codes that would be included in the study. This recommendation rested on the recognition of the local city councilors and mayors as leaders in the African American community who are respected and sought out by their constituents: *“This is respect, to inform them,”* ^(Y1M6W)^ and *“Transparency, when everyone knows what is going on.”* ^(Y2M1S)^ The discussion revealed the need to respect not only the individual participants but also the communities and the culture of the communities from which participants come. One CAB member summed up the discussion. *“I think this is the issue now. People are not connected. That is the problem in [city name]. People feel left out and like you are trying to come in the back door…Everyone likes protocol.”* ^(Y1M4S)^

The discussion on mechanisms for reaching out to the elected officials ranged from written communication to individual meetings to making a presentation at the city council meeting. However, during the discussion of political concerns, logistics, and human resources, the CAB landed on the suggestion of hosting a luncheon in which relevant city council members, the mayors for both cities where the study would take place, the county commissioners, and state representatives from the relevant districts were invited. The CE Team worked with the university’s Office of Governmental Relations and marketing department to draft invitations for the government officials, plan the agenda, and communicate with those to be invited. These plans were vetted by the CAB. As a result, a total of 16 officials received invitations to attend.

The luncheon, held at a university facility, was attended by four of the invitees (25% participation), CAB members, and representatives from both the university and the university health system. University officers and co-principal investigators provided presentations on the research study and the need for and purpose of community engagement. They also responded to questions. Each attendee received a resource packet that included all of the materials and information that would be shared with possible participants once recruitment started. One official – the mayor of a city that is predominantly African American – expressed appreciation for the luncheon and the respect of informing officials.

Two state legislators communicated regret for not being able to attend the function and arranged to meet one-on-one with the co-principal investigator responsible for community engagement, bringing the overall response rate to 37.5%. Although elected official’s participation rate was low, the event marked the first time that a group of high-level academic and political leaders had participated alongside CAB members in a formal introduction of a research study to the community, representing a step forward in the commitment of the University to engagement etiquette. Following the luncheon, the CAB advised ongoing updates to maintain the connection with the government officials who had been introduced to the study. Letters with statistics on recruitment were sent about six months after the luncheon.

### Bidirectional Communication

From the initial CAB meeting, members asked questions about whether or not the research would contribute to the understanding of the impact of environmental factors in their communities. Their comments and questions included stories of their own lived experiences with environmental exposures from local industry and their concerns about the impact on the areas where they live. In a meeting between the CAB and the principal investigator (PI), these questions raised awareness of issues that had not been seen or thought about prior to the project. In that conversation, the PI and the co-Pi for community engagement acknowledged a lack of understanding of the environmental realities in which the potential study participants lived and committed to include this knowledge in their future research considerations.

The CEO of a community-based organization asked for a visit to the lab at the very first CAB meeting. In response, the two CAB groups met for a united Saturday meeting and toured the lab where the collected samples were processed. The PI demonstrated the equipment used in the study and fielded questions from the CAB members. The lab visit created a level of openness and transparency highly welcomed by the CAB members.

## Discussion

The CE Team collaborated with bench scientists to recruit individuals from a historically and intentionally excluded community to participate in a research study focused on DNA damage and repair [17]. Acknowledging the history of research abuses and the need to respectfully engage possible participants, the CE Team enlisted a CAB to play an active role in developing recruitment processes and project materials. Through bidirectional communication, the CAB helped develop a successful process that resulted in the recruitment of 191 people with a basic understanding of the study. Lessons learned in the process included the importance of language, the value of truly accessible materials, and the power of engagement with community leaders.

### Language of Partnership and Respect

Language matters. The CAB forced the CE Team to go beyond using terminology familiar to community members or ensuring that all study materials are written at a level that was accessible for individuals with limited literacy. They infused an awareness and acknowledgment of individuals as valuable members of a community that had faced historic abuses in terms of medical care and research [11]. Study recruitment procedures needed to demonstrate valid reasons why certain areas or individuals are being recruited in a way that does not appear to be targeting historically and intentionally excluded groups as seen in the CAB’s revising of recruitment materials to focus on “Why I am Important” to this study instead of a utilitarian listing of inclusion criteria. This concern was seen in the specific use of the word “targeting” by CAB members when asking about recruitment criteria and how these were explained to possible participants.

The language and posture of those working on research studies address and allay any fears that the research study will take advantage of communities experiencing disadvantage to benefit the majority community [4]. Similar to the findings of Andress, et. al, and Isler, et. al., the CAB showed how community engagement provides a perfect mechanism for evaluating language and pointing out the potential pitfalls and misunderstandings that can arise with the phrasing used by the research team. This includes the uncovering of implicit bias among the research teams [10,31] as well as the failure to see the wording and activities from a different worldview and set of life and community experiences [6].

Concerns over the protection of confidentiality and individual rights points to the importance of directly and transparently addressing the rights of research participants. Including the “Research Subject’s Bill of Rights” as a part of the informed consent discussion creates a space for possible participants to ask their questions about concerns, they have. At the same time, reviewing the rights, as opposed to providing a handout, communicates a deeper level of sincerity for respecting rights. This in turn provides more credence to the guarantees of confidentiality and privacy for those who choose to participate in the research study [10,32,33]. As one CAB member said, “when you talk about rights people feel important.”

### Accessible Recruitment Materials

The CAB pointed out the ways in which specialized terms like DNA, genetic, and genomic were problematic having the potential to create barriers to participation in the study from community members. Language lays the foundation for communication especially when accompanied by an attitude of respect and humility on the part of research team members. As Han et. al. discussed, clarity and transparency in communications forms the basis for trust building [34]. Written materials reflect the same concern and care for communicating well, thoroughly, and transparently. When creating such materials, the partnership of CAB members helps to shine light on phrases, thoughts, or concepts that can be experienced as derogatory or confusing by individuals from historically and intentionally excluded groups.

In this study, the partnership resulted in the development of visual aids to help with the informational and consent processes with possible research participants. Such materials become accessible not simply because they are below grade 8 reading level in the Flesch-Kincaid Readability software [35]. The accessibility is as much related to the relatability based on terminology and concepts used [9] as seen in the discussion of words such as “project” or “study.” The CAB, with members from the communities from which the study drew, play an invaluable role in interpreting the materials created by research teams and helping them use words that affirm the community members while not triggering negative or traumatic reactions because the phrasing had a different connotation than that intended simply because of differences of history and experiences between the research team and the community members they hope to work with [32]. The CE Team experienced this essential CAB role in the discussions on targeting, confidentiality, informed consent, as well as the overt discussions of language.

### Respectful Community Engagement Reaches Beyond Research Participants

Communities that are historically and intentionally excluded have cultures and protocols that influence interactions with outside research teams. The CAB provided a window into this area for engagement to show a deeper level of respect and care for the communities where the research would take place as seen with the planning of a luncheon for public officials from communities in the study area. The communication, and need to make information accessible, goes far beyond the CAB and individuals invited to participate in the research study. In this way, the CAB can serve as an advocate for the community/ies in question. Depending on the cultural norms and expectations for those community members, a research team may need to engage with institutions such as churches, government representatives, or other community-based organizations seen as providing a trusted voice in the community. The CAB in the DNA integrity project played this role by encouraging the provision of information to city leaders from where study participants were to be recruited. While not part of the direct participant recruitment, these interactions, in resonance with the findings from the modified Delphi panels conducted by Khodyakov et. al., demonstrate the desire to authentically engage with the community and provide opportunities to answer questions about the research, acknowledge and allay fears, and explain reasons behind choosing the study area in addressing concerns of targeting groups that are historically and intentionally excluded [36].

## Conclusion

CABs provide a key mechanism for accountability in research. The bidirectional communication between community stakeholders and researchers offers the possibility of enriching not only the research but also the relationships between groups experiencing disadvantages and academic research teams. The CAB-research team relationship creates an atmosphere of learning for both CAB members and researchers. In this way, studies have a higher chance of recruiting diverse populations while also improving community knowledge and understanding around research processes and protections.
